# Exosomes derived from miR-301a-3p-overexpressing adipose-derived mesenchymal stem cells reverse hypoxia-induced erectile dysfunction in rat models

**DOI:** 10.1186/s13287-021-02161-8

**Published:** 2021-01-25

**Authors:** Li Liang, Dachao Zheng, Chao Lu, Qinghong Xi, Hua Bao, Wengfeng Li, Yufei Gu, Yuanshen Mao, Bin Xu, Xin Gu

**Affiliations:** 1 Department of Respiratory Medicine, Shanghai Ninth People’s Hospital, Shanghai Jiao Tong University School of Medicine, Shanghai, 201999 China; 2 Department of Urology, Shanghai Ninth People’s Hospital, Shanghai Jiao Tong University School of Medicine, Shanghai, 201999 China

**Keywords:** Erectile dysfunction, Chronic intermittent hypoxia, Exosomes, miR-301a-3p, Autophagy

## Abstract

**Background:**

Erectile dysfunction (ED) has often been observed in patients with obstructive sleep apnea (OSA). Research on adipose-derived mesenchymal stem cell (ADSC)-derived exosomes has shown that they have significant therapeutic effects in many diseases including ED.

**Methods:**

In this study, ED was induced in Sprague Dawley (SD) rats using chronic intermittent hypoxia (CIH) exposure. CIH-mediated influences were then measured in the corpus cavernous smooth muscle cells (CCSMCs).

**Results:**

Our data showed that miR-301a-3p-enriched exosome treatment significantly recovered erectile function in rats and CCSMCs by promoting autophagy and inhibiting apoptosis. The treatment also significantly recovered the level of alpha smooth muscle actin (α-SMA) in rats and CCSMCs. Bioinformatics predicted that phosphatase and tensin homolog (PTEN) and Toll-like receptor 4 (TLR4) might be targets of miR-301a-3p.

**Conclusions:**

Our results indicate that PTEN-overexpression vectors or TLR4-overexpression vectors reverse the therapeutic effects achieved by miR-301a-3p in CCSMCs indicating that PTEN/hypoxia-inducible factor-1 alpha (HIF-1α) and TLR4 signaling pathways play key roles in the progression of ED. The findings in this study suggest that miR-301a-3p should be considered a new therapeutic target for treating ED associated with OSA.

**Supplementary Information:**

The online version contains supplementary material available at 10.1186/s13287-021-02161-8.

## Introduction

Erectile dysfunction (ED), also known as inadequate penile erection, is a common clinical entity that mainly affects males older than 40 years [1]. It is defined as the inability to achieve and maintain an adequate erection to permit satisfactory sexual intercourse [2] resulting in dissatisfaction with sex life in a significant proportion of men [3]. Several factors are associated with ED including smoking, hormonal imbalance, general health status of the individual, diabetes mellitus, cardiovascular diseases, obstructive sleep apnea (OSA), and psychiatric disorders [4, 5].

Chronic intermittent hypoxia (CIH) is one of the most important and direct consequences of obstructive sleep apnea [6, 7] with studies showing a higher ED incidence in male patients with chronic hypoxia [8]. Hypoxia may affect erectile function, neuronal nitric oxide synthase (nNOS), and endothelial nitric oxide synthase (eNOS) expression leading to erectile dysfunction under hypoxic conditions in murine models [9, 10]. Oral phosphodiesterase type 5 (PDE-5) inhibitors were the first and most effective form of oral therapies recommended for the treatment of ED [11]. However, potential side effects such as anterior ischemic optic neuropathy and increased risk of stroke have hindered the use of PDE-5 inhibitor [12].

Adipose-derived stem cells (ADSCs), originating from the mesoderm of cells, are mesenchymal stem cells with multidirectional differentiation potential [13]. Compared to other stem cells, ADSCs have a lot of advantages such as huge storage, easy to isolate, high speed of proliferation, safe and security, low immunogenicity, and so on [14]. Various studies have shown that ADSC therapy has therapeutic effects for ED resulting from cavernous nerve injury [15–17]. Our previous research also confirmed that ADSC therapy could improve the long-term outcomes in neurogenic, myogenic, and vascular tissue regeneration in the treatment of neurovascular-injury ED [18]. Recently, numerous studies revealed that transplanted stem cells exert treatment effect via paracrine secretion rather than through direct cell replacement [19]. As a class of extracellular vesicles, exosomes play a considerable role in paracrine regulation. Exosomes are membrane vesicles that are secreted by most cells. Exosomes having a diameter of 30–100 nm contain many macromolecular components including proteins, mRNAs, and microRNAs (miRNAs) which can regulate intracellular signaling pathways [20]. As natural vesicles of gene delivery, stem cell-derived exosomes show a wide range of treatment action, which formerly belonged to stem cells [21, 22]. Studies have found that exosomes derived from ADSCs and mesenchymal stem cells (MSCs) exert therapeutic effect on ED in rat models having diabetes and cavernous nerve injury [23–26]. However, despite their great potential in therapeutic delivery, stem cell-derived exosomes have shown limited application in clinical studies because of various difficulties, and the low yield poses a major challenge to further applications [27]. Hence, ADSCs, with the advantages of rapid proliferation and wide distribution in human body, are an ideal type of stem cells producing a large number of exosomes.

miRNAs are short, endogenous, non-coding RNAs (NC RNAs) that represent a part of the genome, do not code for proteins, and play a regulatory role in almost every cellular process through negative control of gene expression [28]. Previous studies have shown that miR-301a-3p is a key factor in pancreatic cancer, breast cancer, carcinoma, and schizophrenia [29–32]. One study indicated that the level of miR-301a-3p in the corpus cavernosum of type 2 diabetes mellitus-associated erectile dysfunction (T2DMED) mice was significantly decreased compared with normal mice [33]. In addition, our preliminary research found that miR-301a-3p was significantly downregulated in the serums of ED patients and rats with CIH-induced ED. This led to our focus on the potential role of miR-301a-3p in the progression of ED. However, miRNAs tend to be easily degraded by RNase in vivo and have a short half-life, which limits their application in treatment [34]. With the development of cell-free transplantation strategy, compared with ADSC, ADSC-derived exosomes are more easily preserved, less easily degraded, and more convenient to transport. Hence, we considered whether ADSC-derived exosomes could be applied as a carrier of miRNA to achieve a combination of their functions and effects. Vast microRNAs are packaged in exosomes and almost 70–80% of circulating RNAs are derived from adipose tissue [35]. These results showed that miRNAs may play a vital role in ADSC-derived exosomes. So, we focus our study on miRNAs contained in ADSC-derived exosomes. Several studies have shown that ADSC-derived exosomes play an important role in ADSC therapy where the use of exosomes from miRNA-modified ADSC to deliver exogenous miRNAs provides protection from various diseases [36–39]. However, there is no report on the treatment of ED by exogenous miRNA-modified ADSC-derived exosomes. Therefore, the study of miRNA-overexpressing (OE) ADSC-derived exosome treatment for ED is vital.

In this study, we used exosomes derived from miR-301-3p-overexpressing ADSC as the therapeutic medium. We analyzed intracavernous pressure (ICP) and arterial pressure (AP) in rat models after CIH exposure. Expression levels of nNOS and eNOS were measured to investigate the potential effects of miR-301a-3p-enriched exosome treatment. Analysis of the expression levels of apoptosis, autophagosomes, autolysosomes, and other indicators associated with ED was done using CIH-exposed murine models. Results showed that miR-301a-3p-enriched exosome treatment had significant therapeutic effects on rats after CIH exposure. Further study of signaling pathways indicated that phosphatase and tensin homolog (PTEN) and Toll-like receptor 4 (TLR4) might be directly targeted by miR-301a-3p with overexpression of both reversing protection effects induced by miR-301a-3p. Our findings indicate that miR-301a-3p should be considered a new therapeutic target in treating ED patients.

## Materials and methods

### Animals

Sprague Dawley (SD) rats (male, weight 180–220 g) were purchased from Shanghai SLAC Laboratory Animal Co., Ltd. Animals were maintained under controlled conditions with a 12/12-h light/dark photoperiod, temperature of 22 ± 3 °C, and humidity of 60 ± 5%. This study was conducted with strict accordance to the Guide for the Care and Use of Laboratory Animals (eighth edition, 2011, published by The National Academies Press, 2101 Constitution Ave. NW, Washington, DC 20055, USA). The protocol was reviewed and approved by the Shanghai Ninth People’s Hospital Institutional Review Board (permit number, HKDL2013001b). Surgery was performed under sodium pentobarbital anesthesia with all efforts being made to minimize suffering.

### Patient samples

A sample size of 30 patients with severe OSA and moderate ED (The International Index of Erectile Function, 5–12) admitted at Shanghai Ninth People’s Hospital, Shanghai, China, was enrolled. According to hospital records, the patients had been clinically diagnosed with ED. The age of the patients ranged from 30 to 65 years and had a diagnosis of severe OSA, as verified by full-night attended polysomnography or polygraphy (i.e., apnea-hypopnea index ≥ 30 per hour of sleep). Patients with hypertension, diabetes, trauma, smoke, using drugs that affect erectile function, BMI > 35, and surgery history were excluded. Thirty healthy people (age-matched) to be used as controls were also recruited from the hospital. Serum samples were taken within 24 h of symptom onset and frozen in liquid nitrogen and stored for short term until further analyses. Ethical approval for the study was provided by the Independent Ethics Committee of Shanghai Ninth People’s Hospital, Shanghai, China. Guidelines from the Ethics Committee were followed where informed and written consent was obtained from all patients or their advisors before samples were collected.

### Culturing ADSCs

Rats ADSCs were collected from the inguinal fat pad. Adipose tissues were washed with phosphate-buffered saline (PBS) to remove residual blood. The tissues were cut into 1-mm^2^ pieces and digested in 1 mg/mL collagenase type II (Sigma-Aldrich, St. Louis, MO, USA) at 37 °C for 1 h followed by centrifugation at 4000×*g* for 5 min. The obtained cell pellet was then suspended in Dulbecco’s modified Eagle’s medium (DMEM) containing 10% fetal bovine serum (FBS), 1% penicillin-streptomycin, and 2 mmol/L l-glutamine. The cells were then cultured in a controlled environment having 5% CO_2_ and a temperature of 38 °C for 48 h. Cells were then transferred into fresh culture medium with subsequent subculture every 3 days. When cells were approximately 90% confluent, they were passaged and used at passage three. For immunofluorescence, cells were then incubated with conjugated monoclonal antibodies against CD29 (ab179471, 1:200), CD44 (ab189524, 1:200), CD90 (ab225, 1:200), CD105 (ab2529, 1:200), and vWF (ab194405, 1:200) (Abcam, Cambridge, UK) at 4 °C for 1 h to confirm the identity of ADSCs. Isotype-identical antibodies (#550343, 1:200, PharMingen) were used as controls. An Operetta High Content Imaging System (Perkin-Elmer, Waltham, MA, USA) was used to obtain the images of the cells. For flow cytometer, cells were identified and selected by flow cytometry (FCM) with anti-CD29, CD44, CD90, and CD105 (1:200; Abcam). After being subcultured to the third generation, cells at 80% confluence were washed twice with PBS followed by digestion with 0.25% trypsin-ethylenediaminetetraacetic acid (EDTA) (Thermo Fisher, MA, USA). The cells were then centrifuged at 1000 rpm and washed with PBS. After incubation with antibodies and their isotype controls (1:200) (PharMingen, CA, USA) at 4 °C for 30 min, the cells were flowed through the cytometer (Becton Dickinson, Franklin Lakes, NJ, USA) at about 1000 cells per second and analysis.

### Isolation of exosomes

Adipose-derived stromal cells collected from miR-301a-3p mimic (ADSCs transfected with miR-301a-3p overexpressing mimic), control (untreated ADSCs), and miR-NC (ADSCs transfected with miRNA mimic negative control) groups at 80–90% confluence were washed with PBS and cultured in microvascular endothelial cell growth medium-2 media deprived of FBS. ADSCs were then supplemented using 1× serum replacement solution (PeproTech) for 24 h. Dead cells and debris were removed by centrifugation of ADSCs at 300×*g* for 10 min and 2000×*g* for 10 min followed by mixing 10 mL of the supernatant with 5 mL of ExoQuick-TC reagent (System Biosciences). The mixture was then centrifuged at 1500×*g* for 30 min, with the resulting exosome-containing pellet being re-suspended in nuclease-free water. TRIzol-LS (Invitrogen, CA, USA) and Exosomal Protein Extraction (Invitrogen) kits were used for extracting total RNA and protein, respectively. Isolated exosomes were used immediately for experiments or stored at − 180 °C. For transmission electron microscopy (TEM) observation, exosomes were stored in 1% paraformaldehyde, dehydrated via an ethanol series, and embedded in EPON. Sections (65 nm) were stained with uranyl acetate and Reynold’s lead citrate and examined with a transmission electron microscopy (CM-120 electron microscope, Philips). The specific exosome markers, including CD9, CD63, and TSG101 were identified by Western blot analysis.

### CIH exposure-induced ED rat model

Twenty-four male SD rats were randomly divided into control, CIH, CIH + exosomes from untreated ADSCs (Exo), and CIH + exosomes from miR-301a-3p overexpressing ADSCs (Exo-301a) groups (*n* = 6). An oxygen sensor was placed at the bottom of the chamber to measure the oxygen content in the CIH exposure chamber over the course of several cycles. Animals were exposed to 2 min of 5% O_2_ for each 4-min cycle with each challenge lasting 8 h. The challenge was done for 8 weeks during the daytime from 8 am to 4 pm. Sham group rats were exposed to 21% O_2_. Exosomes (400 μg of protein) were isolated using 200 μL PBS and then administered using intracavernous injection for Exo groups, whereas control rats received an equal volume of PBS. Exosomes were administered to the rats every week for 8 weeks.

### Erectile function measurement

After 8 weeks of CIH exposure, the intracavernous pressure (ICP) and real-time carotid arterial pressure (RT-AP) were recorded simultaneously as described in our previous article [40]. In brief, while under anesthesia, the right carotid artery, crus penis, and bilateral cavernous nerves were exposed. Two 25-gauge catheters, filled with 250 U/mL heparin solution and connected to a pressure transducer (Labchart, Colorado Springs, USA), were separately inserted into the carotid artery and crus penis to record the RT-AP and ICP simultaneously. Using an electrode hook, we stimulated one side cavernous nerve at intervals of 5 min (3 times/side). Stimulation parameters were 1.5 mA, 20 Hz, pulse width 0.2 ms, and duration 60 s. The maximum ICP (MICP) and RT-AP of unilateral stimulation was selected for calculating mean ICP and mean AP of each rat. After stimulation, the penis was divided into two parts. One part was frozen in liquid nitrogen for Western blot, and another one was fixed for histologic analysis.

### Immunofluorescence

Harvested tissues were immersed in optimal cutting temperature compound and immediately frozen in liquid nitrogen. The tissues were fixed in 4% paraformaldehyde, embedded in optimal cutting temperature, and cut into sections having a thickness of 5 μm followed by immunofluorescence staining as described in [18]. Primary antibodies used in this study were eNOS (ab76198), nNOS (ab76067), and Phalloidin (ab176753) all obtained from Abcam at 37 °C for 2 h. Secondary antibodies included Alexa-488, Texas Red-conjugated antibodies (1:500; Invitrogen, CA, USA), and Texas Red goat anti-rabbit IgG (1:200; Life Technologies, Grand Island, NY, USA). Nuclei were stained using 4′,6-diamidino-2-phenylindole (DAPI) (1:10,000, Invitrogen, CA, USA). The number of cells in each image was counted with DAPI cells and positive cells. Ratio of positive nNOS counts to DAPI was used to analyze the nerve fibers in the dorsal section of the penis. Smooth muscle and endothelial stains were analyzed using the ratio of positively stained areas of phalloidin and eNOS to DAPI in the corpora cavernosa.

### CCSMC culture and CIH exposure

Rats were sacrificed where on a sterile table, the penis was excised and placed in a sterile Petri dish followed by two washes using PBS. The skin around the penis was carefully peeled away, along with the albuginea, urethral sponge, cavernous body, and other vessels. The corpus cavernosum was cut into 1-mm^3^ tissue blocks that were placed in a cell culture flask containing 0.5% type I collagenase solution (Sigma). Cells were cultured at 37 °C with shaking in a humidified atmosphere having 95% air and 5% CO_2_ for 3 h. The cells were then filtered and centrifuged followed by the addition of 3 mL F12 medium (Invitrogen) containing 20% fetal bovine serum (Invitrogen) and incubated at 37 °C and 5% CO_2_. Long, spindle-shaped SMCs were observed at the bottom of the 25-cm^2^ culture flasks after incubating for 24 h. For CIH exposure, corpus cavernous smooth muscle cells (CCSMCs) were exposed to 5 min of 14 to 15% O_2_ during each 60-min cycle for 24 h by using BioSpherix-OxyCycler C42system (BioSpherix, Redfield, NY). All cells were cultured for 24 h followed by co-culturing with miR-301a-3p-enriched exosomes for 48 h.

### Statistical analysis

Results are expressed as the mean ± SD. All the data obtained from this study was analyzed using GraphPad 9.0. Two group analysis was performed with *t* test (two tailed). One-way ANOVA was used among various groups with *p* < 0.05 being considered statistically significant.

More detailed materials and methods are in the Supplementary Methods.

## Results

### CIH exposure significantly downregulates miR-301a-3p in patients with ED and in rats induced with ED

**Fig. 1:**
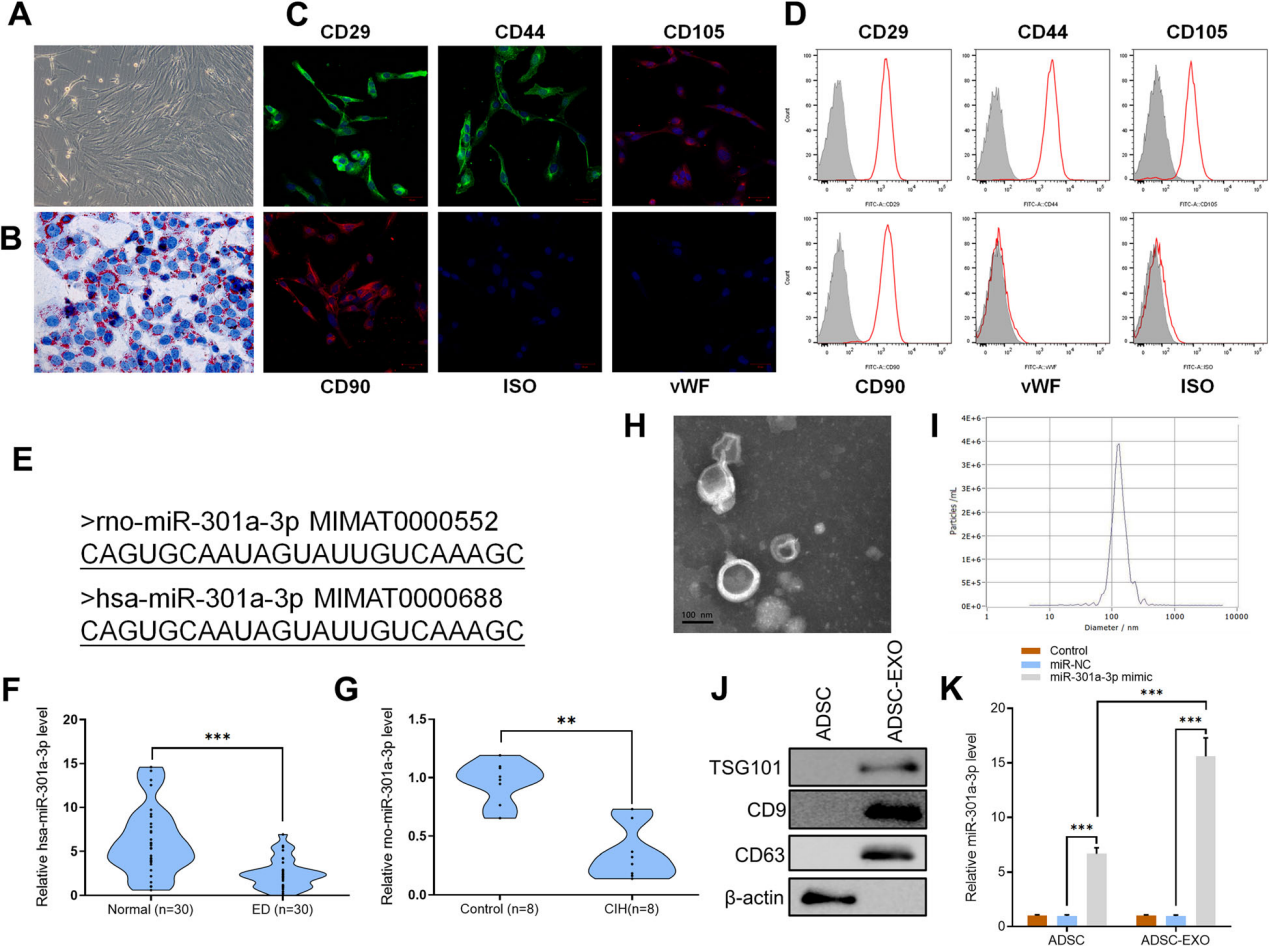
**a** ADSCs were collected from adipose tissues of SD rats. ADSCs displayed a typical cobblestone-like morphology under a microscope. **b** Adipose cells were confirmed with Oil Red O staining. **c** ADSCs were positive for the mesenchymal stem cell (MSC) markers CD29, CD34, CD44, CD90, and CD105. **d** Flow cytometry analysis of the surface markers in ADSCs. **e** Sequence of rno-miR-301a-3p and has-miR-301a-3p. **f** The expression levels of has-miR-301a-3p in serum samples from patients (*n* = 30, ***p < 0.001). **g** The expression levels of rno-miR-301a-3p in serum samples from SD rats (*n* = 8, **p < 0.01). **h** Transmission electron microscopy analysis of exosomes secreted by ADSCs (scale bar = 100 nm). **i** The particle size of the exosomes secreted by ADSCs was measured by nanoparticle tracking analysis. **j** Protein levels of TSG101, CD9, and CD63 in ADSC and ADSC-derived exosomes as determined by Western blotting. **k** RT-qPCR results of miR-301a level in ADSC and ADSC-derived exosomes. Data are expressed as mean ± SD (*n* = 3 for ADSC analysis; ***p < 0.001)

ADSCs obtained from adipose tissues of SD rats displayed a typical fibroblastic-like morphology under the microscope (Fig. 1a). Oil Red O staining confirmed that they were undergoing adipogenesis (Fig. 1b). To confirm the identity of ADSCs, they were incubated with conjugated monoclonal antibodies against CD29, CD44, CD90, CD105, CD34, and vWF with isotype-identical antibodies (PharMingen) being used as controls. Immunofluorescence and flow cytometer results showed that ADSCs were positive for the mesenchymal stem cell (MSC) markers CD29, CD34, CD44, CD90, and CD105 (Fig. 1c, d). The sequences between hsa-miR-301a-3p (human) and rno-miR-301a-3p (rat) were the same (obtained from http://www.mirbase.org/) (Fig. 1e). It was difficult to perform invasive test on human’s corpus cavernosum. Hence, we detected the level of hsa-miR-301a-3p in serum of patients which was a non-invasive test and quantitative reverse transcription polymerase chain reaction (RT-qPCR) analysis of serum samples collected from 30 ED patients showed that expression levels of hsa-miR-301a-3p in ED patients were significantly lower than those in healthy patients (*n* = 30, *p* < 0.001) (Fig. 1f). To further determine the expression of rno-miR-301a-3p in rats’ serum, CIH exposure was done on SD rats. To ensure the consistency of the results, rno-miR-301a-3p expression in the serum of rats was also detected. Results showed that rno-miR-301a-3p expression in rats’ serum was inhibited at gene level in CIH-exposed rats compared to the control group (*n* = 8, *p* < 0.01) (Fig. 1g).

ADSC-derived exosomes were isolated and the morphology of exosomes was observed under a transmission electron microscope (TEM) which exhibited a round-shaped morphology (Fig. 1h). Nanoparticle tracking analysis (NTA) shows that the diameter of most exosomes was approximately 100 nm (Fig. 1i). Examination of ADSCs and exosomes using Western blot resulted in exosomes testing positive against exosome markers TSG101, CD9, and CD63 while ADSCs tested negative (Fig. 1j). After transfection with miR-301a-3p-overexpressing mimic, ADSCs and ADSC-derived exosomes were analyzed using RT-qPCR. Results confirmed the overexpression of miR-301a-3p in both ADSCs and ADSC-derived exosomes, compared to control and miRNA mimic negative control (miR-NC) groups (Fig. 1k). The results indicated that miR-301a-3p was significantly downregulated in ED patients and CIH exposure rats. In addition, miR-301a-3p mimic had good transcription efficiency in ADSCs.

### CIH exposure negatively influences erectile function while miR-301a-3p-enriched exosome (Exo-301a) treatment repairs the damage in SD rats

**Fig. 2:**
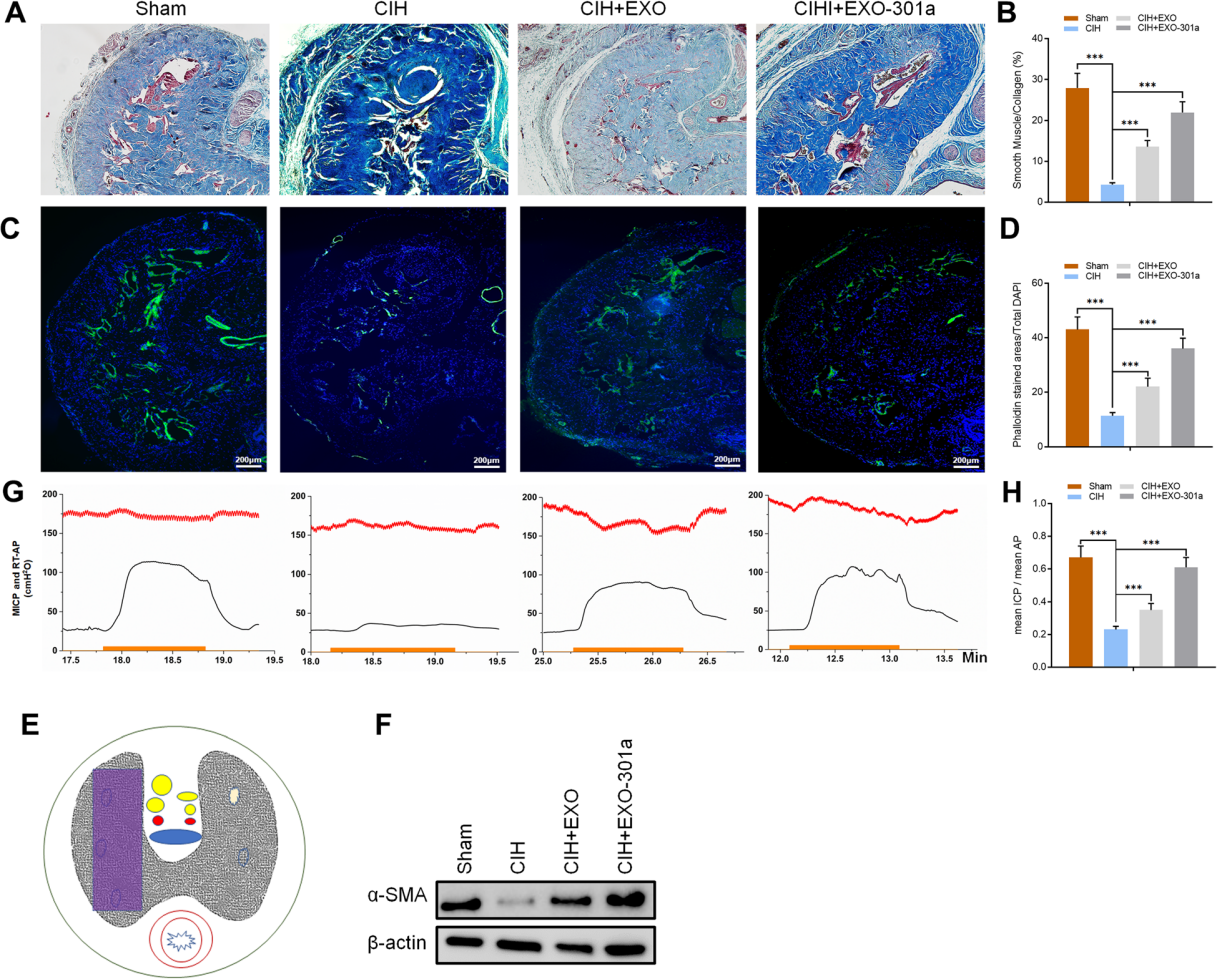
Rats were divided into 4 groups: sham, CIH exposure, CIH + exosomes from untreated ADSCs (Exo), and CIH + miR-301a-3p-enriched exosomes (Exo-301a). **a**, **b** Results of Masson trichrome staining for actin (red) and collagen (blue). **c**, **d** Results of Phalloidin (green) and DAPI (blue) staining in SD rats. **e** The purple rectangle denotes the area of the penis selected for an area for histology analysis. **f** Protein levels of α-SMA in sham, CIH, CIH+EXO, and CIH+EXO-301a groups as determined by Western blotting. **g**, **h** Results of ICP and RT-AP measurement in all four groups. The ICP is indicated with a green curve. The red curve denotes the real-time AP during electrostimulation. The orange bar denotes the 60-s cavernous nerve electrical stimulation. Data are expressed as mean ± SD (*n* = 6; ***p < 0.001)

Masson trichrome staining of actin and collagen was done for each group where smooth muscle and connective tissue in the corpus cavernosum stained red and blue, respectively. Results indicated a decrease in the proportion of smooth muscle when CIH exposure rats were compared with the sham group after (*p* < 0.001). When compared with the CIH exposure group, miR-301a-3p-enriched exosome treatment significantly promoted the proportion of smooth muscle indicating that exosome treatment had therapeutic effects on the repair of smooth muscle (*p* < 0.001) (Fig. 2a, b). Results obtained after Phalloidin staining indicated that CIH exposure destroyed F-actin. Significantly more stained cytoskeleton area was observed after normal exosome treatment and miR-301a-3p-enriched exosome treatment with the effects of the latter being more pronounced (Fig. 2c, d).

The ratio of ICP/RT-AP was used to assess erectile function (Fig. 2e) with results showing that CIH exposure significantly inhibited erectile function in SD rats (Fig. 2g). Normal exosomes and miR-301a-3p-enriched exosome treatments had significant effects on recover ICP/RT-AP when compared to the CIH exposure group (*p* < 0.001) with miR-301a-3p-enriched exosomes having more pronounced effects (Fig. 2g). Western blot analysis was done to measure the level of myofibroblast formation with results indicating that alpha smooth muscle actin (α-SMA) was downregulated after CIH exposure. However, exosome treatment significantly increased the expression of α-SMA when compared with the CIH exposure group (Fig. 2f).

**Fig. 3:**
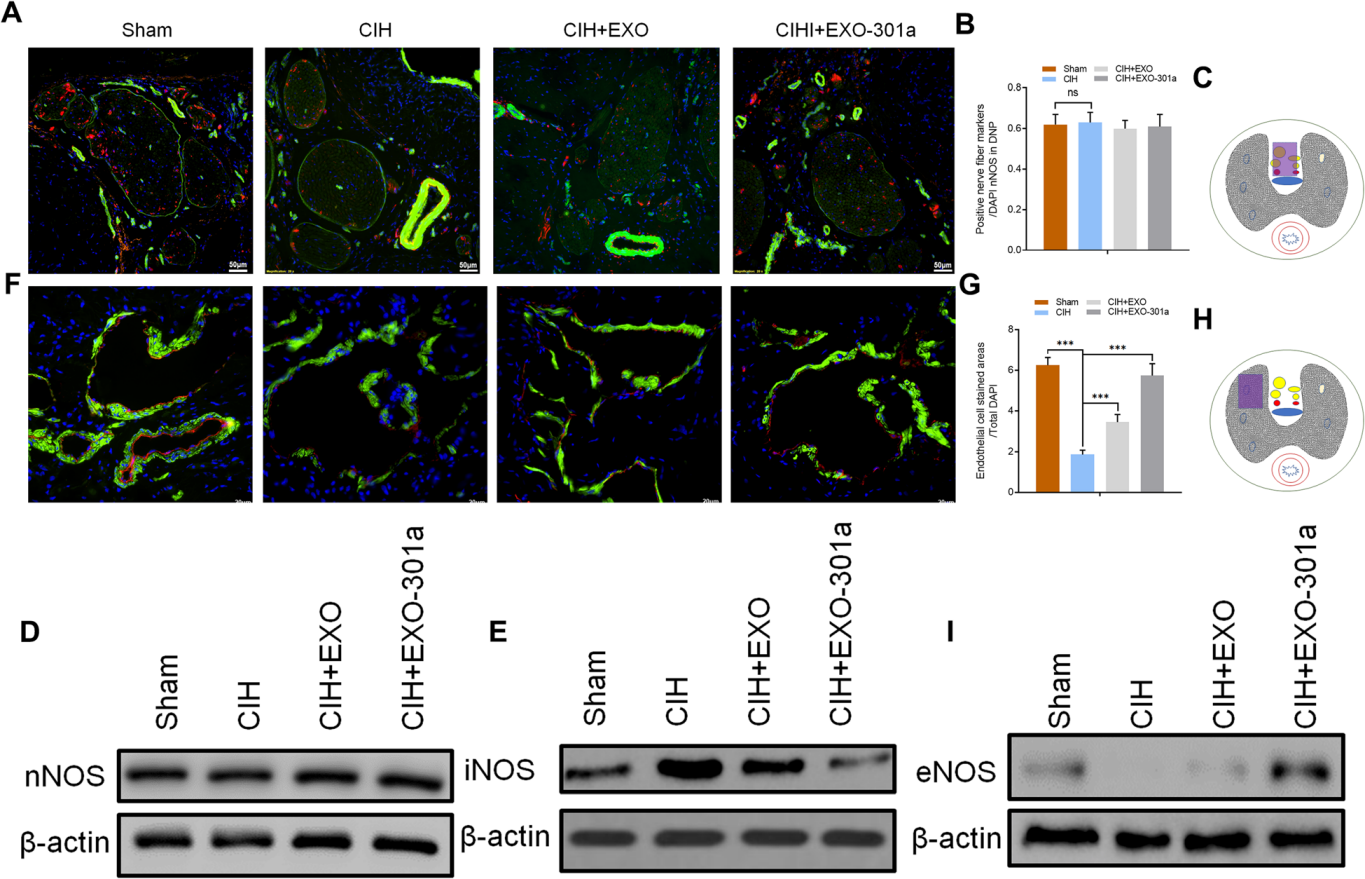
Rats were divided into 4 groups: sham, CIH exposure, CIH + exosomes from untreated ADSCs (Exo), and CIH + miR-301a-3p-enriched exosomes (Exo-301a). **a**–**c** The DNP area selected for nNOS (red) analysis and results of Phalloidin (green) and DAPI (blue) staining. The purple rectangle denotes the area of the penis selected for an area for histology analysis. **d**, **e** Protein levels of nNOS and iNOS in DNP as determined by Western blotting. **f**–**h** The area selected for eNOS (red) analysis and results of Phalloidin (green) and DAPI (blue). The purple rectangle denotes the area of the penis selected for analysis. **i** Protein levels of eNOS as determined by Western blotting. Data are expressed as mean ± SD (*n* = 6; **p < 0.01, ***p < 0.001; ns, non-significant)

To determine the level of nNOS in the dorsal nerve of the penis (DNP), harvested tissues were prepared for immunofluorescence staining and Western blot analysis. Results showed no significant changes in the ratio of nNOS-positive nerve counts/DAPI in all areas of CIH exposure groups when compared with sham groups indicating that CIH exposure did not alter NO release from peripheral nerve endings (Fig. 3a–c). This was confirmed by the results of Western blot analysis which indicated that CIH exposure had no effect on the expression of nNOS. Interestingly, CIH exposure stimulated the expression of inducible nitric oxide synthase (iNOS), while miR-301a-3p-enriched exosomes reduced its expression (Fig. 3d, e). Immunofluorescence staining of endothelial cells showed that eNOS expression decreased significantly after CIH exposure (*p* < 0.001) when compared to the sham group. Exosome treatment had positive effects on recovering the expression level of eNOS with miR-301a-3p-enriched exosome treatment having significantly better results (Fig. 3f–h). Results indicate that CIH exposure negatively affected erectile function while miR-301a-3p-enriched exosome treatment had significant remediation effects on SD rats, including the ratio of mean ICP and mean AP and expression levels of α-SMA and eNOS.

### miR-301a-3p suppressed the level of PTEN and TLR4 in vivo

**Fig. 4:**
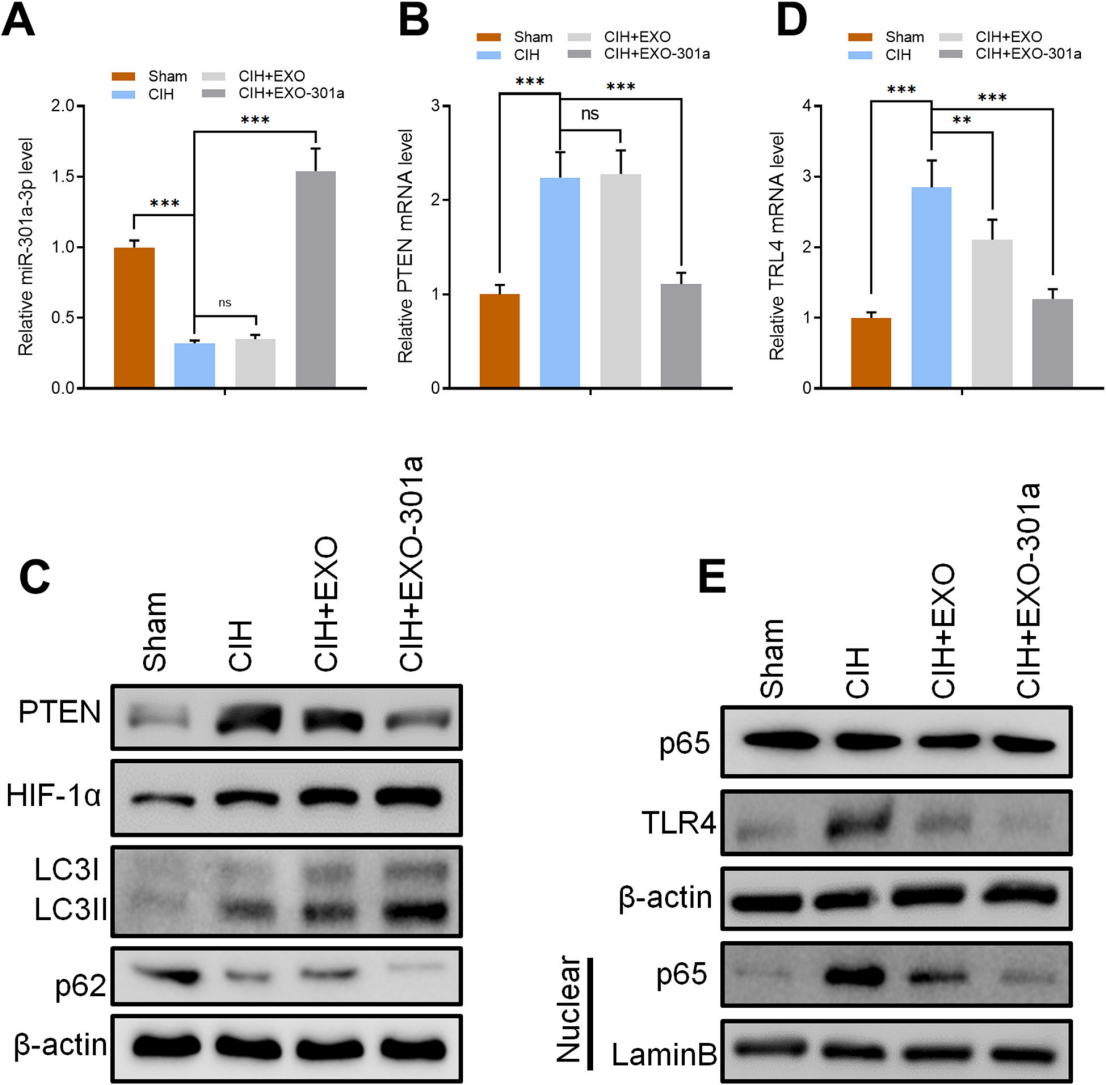
Rats were divided into 4 groups: sham, CIH exposure, CIH + exosomes from untreated ADSCs (Exo), and CIH + miR-301a-3p-enriched exosomes (Exo-301a). **a**, **b**, **d** RT-qPCR results of miR-301a-3p, PTEN, and TLR4 in sham, CIH, CIH+EXO, and CIH+EXO-301a in DNP. **c**, **e** Protein levels of PTEN, TLR4, HIF-1α, LC3I/II, p62, and p65 in DNP as measured by Western blotting. Data are expressed as mean ± SD (*n* = 6; **p < 0.01, ***p < 0.001; ns, non-significant)

DNP tissue were collected from SD rats in all groups (Sham, CIH, CIH+EXO, CIH+EXO-301a) and analyzed using RT-qPCR to determine the signaling pathway used by miR-301a-3p to influence erectile function. RT-qPCR results showed that the expression level of miR-301a-3p in rat DNP tissue significantly decreased in the CIH exposure group when compared to the sham group (*p* < 0.001). There was no significant difference between the CIH exposure group and the CIH+EXO group, while miR-301a-3p was significantly overexpressed in the CIH+EXO-301a group (Fig. 4a). Furthermore, the results showed a significant increase of PTEN and TLR4 gene levels in CIH and CIH+EXO groups (Fig. 4b, d). Treatment with miR-301a-3p reversed the expression of PTEN and TLR4 leading to a decrease in PTEN and TLR4 levels (Fig. 4b, d). Protein levels of PTEN and TLR4 in rat DNP tissue in each group were confirmed using Western blot analysis (Fig. 4c, e).

Results obtained after Western blot analysis in rat DNP tissue showed that CIH exposure directly induced overexpression of LC3I/II and p65 in the nucleus (Fig. 4c, e) indicating that the level of autophagy was upregulated by CIH exposure. Upregulated autophagy was also confirmed by the inhibited expression of p62 (Fig. 4c). Exosome treatment increased the level of autophagy through overexpressing LC3I/II and p65 while the levels of p62 decreased with miR-301a-3p-enriched exosomes having a more pronounced effect (Fig. 4c, e). These results suggest that PTEN and TLR4 can be directly targeted by miR-301a-3p.

**Fig. 5:**
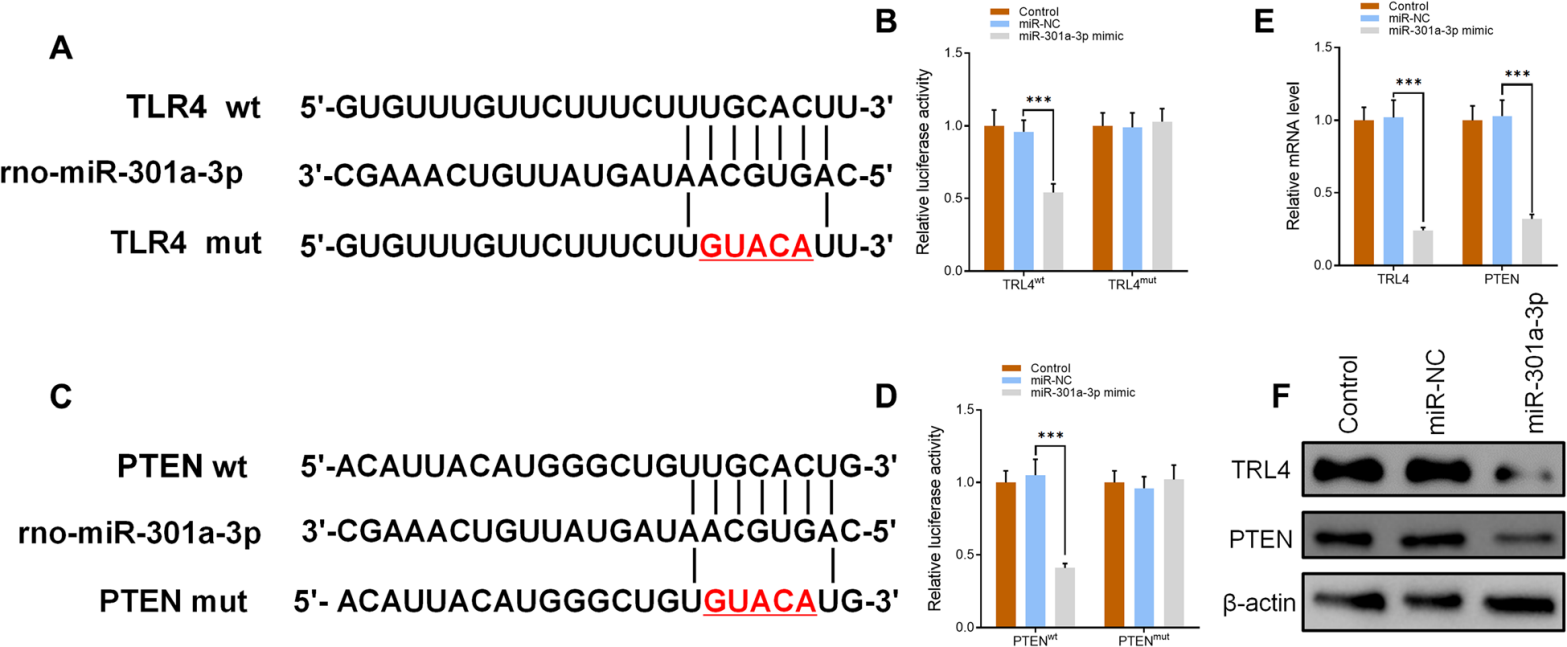
**a**, **c** Results of rno-miR-301a-3p, TLR4 (wt and mut), and PTEN (wt and mut) sequencing. **b**, **d** Luciferase assay of TLR4 (wt and mut) and PTEN (wt and mut) transfected with miR-301a-3p mimic. **e** Expression levels of TLR4 and PTEN in control, miR-NC, and miR-301a-3p groups as measured by RT-qPCR. **f** Protein levels of TLR4 and PTEN in the three groups as measured by Western blotting. Data are expressed as mean ± SD (*n* = 6; ***p < 0.001)

Bioinformatics was used to predict the possible targets in the determination of the potential association between miR-301a-3p and PTEN/TLR4 with results showing that both PTEN and TLR4 could be possible targets of miR-301a-3p (Fig. 5a, c). Dual-luciferase reporter assay results showed that overexpression of miR-301a-3p reduced the intensity of fluorescence in CCSMCs transfected with TLR4-wild type (WT) and PTEN-WT vectors while having no effect on CCSMCs transfected with TLR4-mutant type (MUT) and PTEN-MUT vectors (Fig. 5b, d). RT-qPCR and Western blot results further confirmed that both TLR4 and PTEN were inhibited at mRNA and protein level after cells were transfected with miR-301a-3p (Fig. 5e, f). Combining both sets of results made a clear indication that both PTEN and TLR4 are direct targets of miR-301a-3p.

### miR-301a-3p-enriched exosomes inhibit CIH-induced apoptosis and upregulate CIH-induced overexpression of autophagy in CCSMCs

**Fig. 6:**
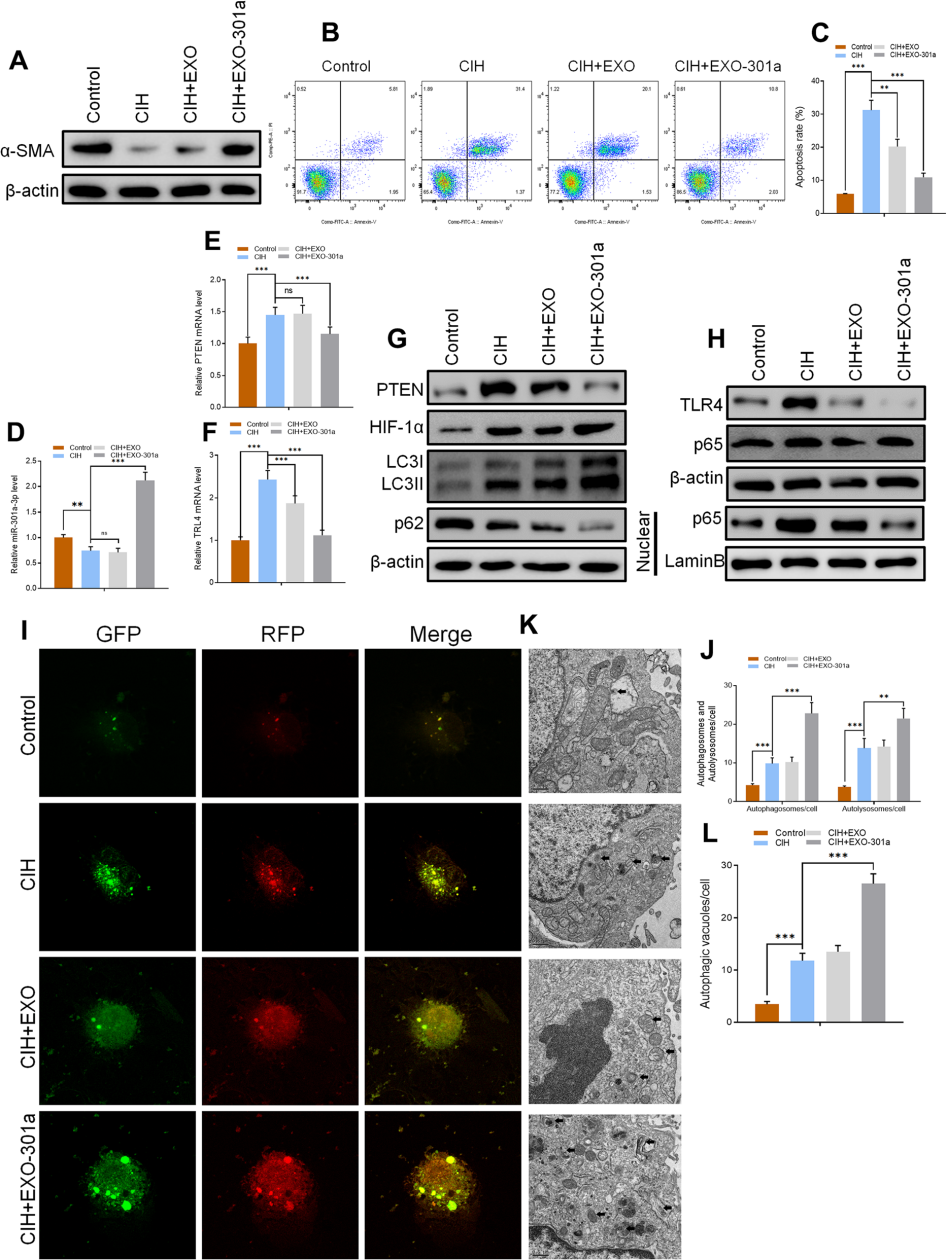
**a** Protein levels of α-SMA in control, CIH, CIH+EXO, and CIH+EXO-301a groups as measured by Western blotting. **b**, **c** Flow cytometry results of apoptosis rate in all groups. **d**, **e**, **f** Relative expression of miR-301a-3p, PTEN, and TLR4 level as determined by RT-qPCR. **g**, **h** Protein levels of PTEN, TLR4, HIF-1α, LC3I/II, p62, and p65 in CCSMCs as quantified by Western blotting. **i**–**l** Results of mRFP-GFP-LC3 staining and quantitation of autophagosomes, autolysosomes, and autophagic vacuoles. Data are expressed as mean ± SD (*n* = 6; **p < 0.01, ***p < 0.001; ns, non-significant)

For CIH exposure, CCSMCs were exposed to 5 min of 14 to 15% O_2_ during every 60 min cycle for 24 h. All cells were cultured for 24 h and co-culturing with miR-301a-3p-enriched exosomes for 48 h. Results obtained after CIH exposure of CCSMCs indicated that α-SMA was downregulated at protein level. On the other hand, exosome treatment increased the level of α-SMA after CIH exposure with miR-301a-3p-enriched exosome treatment having a more significant effect than normal exosome treatment (Fig. 6a). Flow cytometry with Annexin V-Fluorescein isothiocyanate (FITC) staining was used to assess the apoptosis rate with results showing that CIH exposure directly led to a significant increase in the apoptosis rate. However, exosome treatment inhibited apoptosis with miR-301a-3p-enriched exosome treatment having a significantly higher CIH-induced apoptosis rate inhibition than normal exosome treatment (*p* < 0.001) (Fig. 6b, c). Levels of miR-301a-3p, PTEN, and TLR4 were analyzed using RT-qPCR. As we had hypothesized, results indicated that miR-301a-3p levels decreased after CIH exposure (*p* < 0.01) when compared to the control group. There was no significant difference between the CIH group and the CIH+EXO group (Fig. 6d). However, miR-301a-3p-enriched exosome treatment led to a significant overexpression of miR-301a-3p levels in CCSMCS after CIH exposure (Fig. 6d). Both PTEN and TLR4 levels increased significantly after CIH exposure while miR-301a-3p-enriched exosome treatment significantly decreased the mRNA expression level of PTEN and TLR4 (Fig. 6d). Results obtained after Western blot analysis confirmed the expression levels of PTEN and TLR4 (Fig. 6g, h).

In addition, Western blot results showed that CIH exposure directly induced overexpression of LC3I/II and p65 in the nucleus indicating that the level of autophagy was upregulated by CIH exposure (Fig. 6g, h). Increased autophagy was confirmed by the inhibited expression of p62 (Fig. 6g). The level of autophagy was further increased by exosome treatment, especially treatment with miR-301a-3p-enriched exosomes (Fig. 6g, h). Autophagic flux analysis was further done where CCSMCs were transfected with mRFP-GFP-LC3 with results showing that the quantity of autophagosomes, autolysosomes, and autophagic vacuoles increased significantly after CIH exposure (Fig. 6i–l). There was no significant difference between the CIH group and the CIH+EXO group, while miR-301a-3p led to a significant increase of autophagosomes, autolysosomes, and autophagic vacuoles in CCSMCs (Fig. 6i–l). Our findings suggest that miR-301a-3p-enriched exosome treatment inhibits CIH-induced apoptosis and upregulates CIH-induced overexpression of autophagy in CCSMCs.

### PTEN or TLR4 overexpression significantly suppresses exo-301a-3p-induced positive effect on autophagy and inhibitory effect on apoptosis

**Fig. 7:**
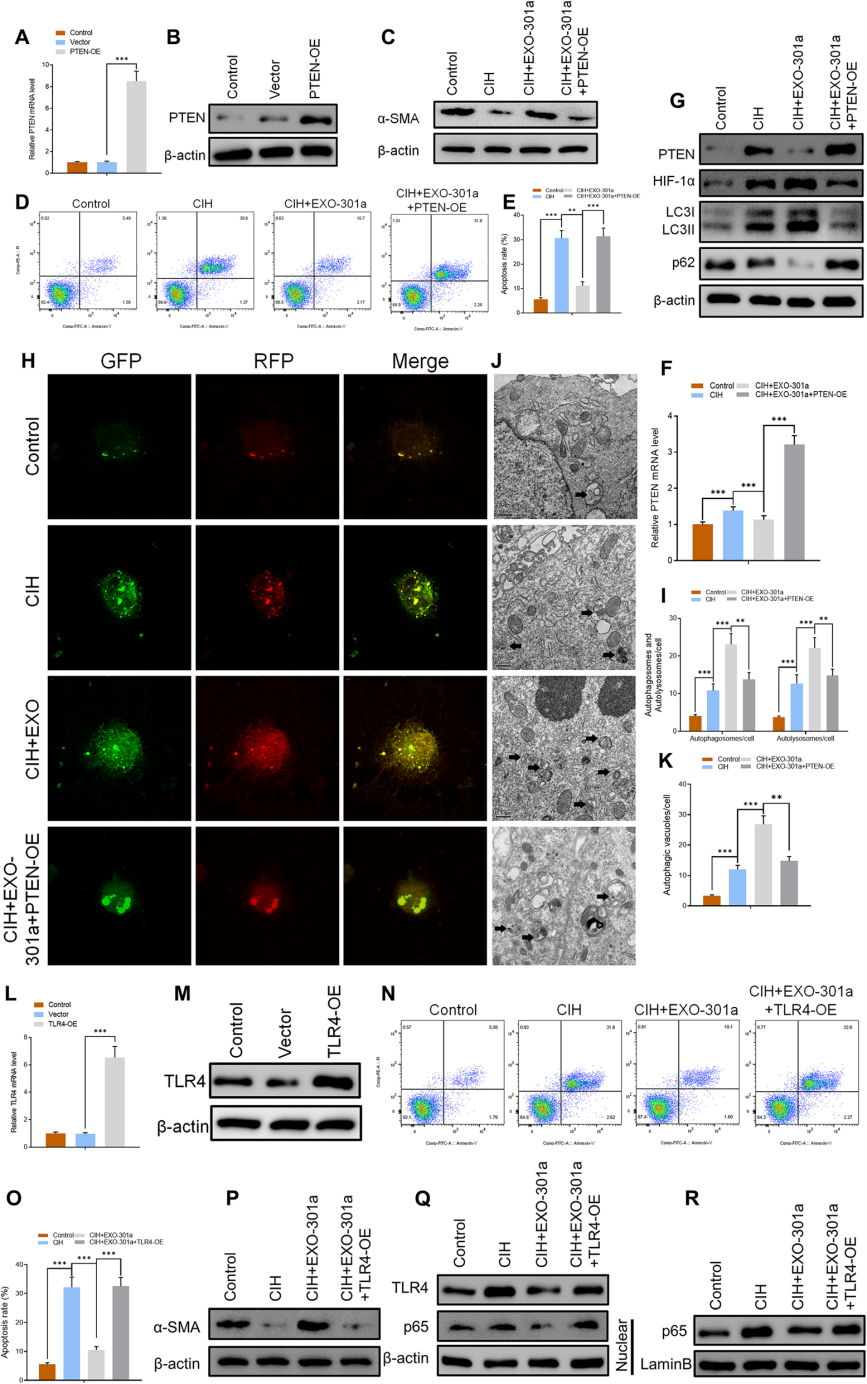
**a**, **b** Relative mRNA and protein level of PTEN in control, vector, and PTEN-OE groups. **c** Protein levels of α-SMA in control, CIH, CIH+EXO, and CIH+EXO-301a groups as measured by Western blotting. **d**, **e** Apoptosis rate in four groups as determined by flow cytometry. **f** RT-qPCR results of PTEN in four groups (***p < 0.001). **g** Protein level of PTEN, HIF-1α, LC3I/II, and p62 as assessed by Western blotting. **h**–**k** mRFP-GFP-LC3 staining and quantitative results of autophagosomes, autolysosomes, and autophagic vacuoles in the four groups. **l**, **m** Relative mRNA and protein level of TLR4 in control, vector, and TLR4-OE groups. **n**, **o** Level of apoptosis in control, CIH, CIH+EXO, and CIH+EXO-301a groups. **p**–**r** Protein levels of α-SMA, TLR4, and p65 as measured by Western blotting. Data are expressed as mean ± SD (*n* = 6; **p < 0.01, ***p < 0.001)

PTEN-overexpressing (PTEN-OE) and TLR4-overexpressing (TLR4-OE) vectors were constructed to determine whether miR-301a-3p/PTEN/TLR4 signaling pathways were involved in the progression of apoptosis and autophagy. After transfection, overexpression of PTEN was detected using RT-qPCR and Western blot analysis (Fig. 7a, b). Western blot results confirmed that miR-301a-3p reversed the CIH-induced suppressive effects on α-SMA while PTEN-overexpressing (OE) inhibited the expression level of α-SMA in the CIH+EXO-301a+PTEN-OE group (Fig. 7c). On the other hand, miR-301a-3p-enriched exosome treatment resulted in a CIH-induced increase of HIF-1α and LC3I/II levels while at the same time inhibiting the expression of p62 (Fig. 7g). All miR-301a-3p-induced effects on levels of HIF-1α, LC3I/II, and p62 were reversed by PTEN-OE (Fig. 7g). Flow cytometry results indicated that CIH exposure led to a significantly high apoptosis rate with the effects promoting apoptosis being suppressed by miR-301a-3p. However, PTEN-OE reversed the miR-301a-3p-induced inhibitory effects on apoptosis (Fig. 7d, e). In addition, autophagic flux analysis confirmed that CIH-induced increase of autophagosomes, autolysosomes, and autophagic vacuoles after miR-301a-3p-enriched exosome treatment (Fig. 7h–k). However, transfection with PTEN-OE significantly decreased the quantity of autolysosomes and autophagic vacuoles in CCSMCs.

Potential roles of TLR4 were also determined where the effectiveness of TLR4-OE was checked at protein and gene level (Fig. 7l, m). Flow cytometry results showed that the high CIH-induced apoptosis rate was inhibited by miR-301a-3p. TLR4-OE significantly suppressed miR-301a-induced inhibitory effects thereby promoting increased apoptosis (Fig. 7n, o). Similar to results of the CIH+EXO-301a+PTEN-OE group, miR-301a-induced expression of high α-SMA levels was significantly suppressed by TLR4-OE vectors (Fig. 7p). In addition, Western blot analysis confirmed that TLR4-OE reversed the miR-301a-3p-inhibted expression level of p62 in CCSMCs (Fig. 7q, r). When combined, the results suggest that both PTEN-OE and TLR4-OE significantly suppressed miR-301a-3p-induced positive effects on autophagy and inhibitory effects on apoptosis.

## Discussion

There is an increase in the incidences of prostate cancer in line with increasing male life expectancy. Despite the use of nerve-sparing techniques during the treatment of prostate cancer, rates as high as 90% of post-robotic-assisted laparoscopic radical prostatectomy (RALP) ED have been reported [40]. Other important risk factors associated with ED are CIH and sleep apnea problems in men [41]. Traditionally, clinical interventions have been limited to managing the chronic form of ED using phosphodiesterase-5 inhibitors, intracavernosal injections, vacuum devices, and penile prostheses. Recently, exosomes have been found to have therapeutic effects on ED in rat models having diabetes and cavernous nerve injury [22, 24, 25]. In this study, we designed experiments to investigate the effects of CIH exposure in rat models and CCSMCs. The main aim of the study was to determine the role that miR-301a-3p plays in CIH exposure rats and CCSMCs as well as determining its molecular mechanisms.

In the treatment of ED, cavernous smooth muscle plays a key role in recovery of erectile function [42]. Our results indicated that miR-301a-3p-enriched exosome treatment significantly increased the proportion of smooth muscle in CIH exposure rats. Moreover, miR-301a-3p-enriched exosomes led to a significantly raised expression of α-SMA. Erectile function recovery was also determined by measuring ICP and RT-AP levels. Generally, both exosome treatments had significant effects on recover ICP/RT-AP in CIH exposure rats with miR-301a-3p-enriched exosome treatment having better therapeutic effects. Production of NO, a key factor in erectile function, was determined by examining the levels of nNOS and eNOS. Results showed that the expression level of eNOS in DNP and sinusoid decreased significantly in CIH exposure groups when compared with sham groups. On the other hand, miR-301a-3p-enriched exosome treatment led to a significantly higher expression of eNOS in CIH exposure rats. Results from this study indicated that miR-301a-3p-enriched exosomes have therapeutic effects on recovering erectile function damaged after CIH exposure. Besides, RT-qPCR data showed that the levels of miR-301a-3p in ED with CIH patients were significantly lower than those in healthy patients in clinical levels. The stability of miRNA of serum stored at − 80° in the short term did not decrease significantly [43]. But for the accuracy of the experiment, we will expand the sample size in future study, detect miRNA expression in serum of more patients, and further investigate the effect and mechanism of miR-301a-3p as clinical marker and therapeutic target.

A previous study recommends using MSC-induced promotion of autophagy to treat ED [44]. In this study, we examined the expression levels of LC3I/II, p62, and p65. Results showed that autophagy was stimulated to a higher level in CIH exposure rats and CCSMCs. miR-301a-3p-enriched exosome treatment increased the number of autophagosomes, autolysosomes, and autophagic vacuoles in rats and CCSMCs after CIH exposure. The level of apoptosis was assessed using flow cytometry after Annexin V-FITC staining. We found that CIH exposure increased the apoptosis rate in CCSMCs, while miR-301a-3p reversed these effects by decreasing the apoptosis rate. Our findings supported the prediction by bioinformatics that PTEN and TLR4 could be targets of miR-301-3p. Therapeutic effects of miR-301a-3p-enriched exosome treatment on the levels of HIF-1α, α-SMA, autophagy, and apoptosis were reversed by both PTEN-OE and TLR4-OE. These findings suggest that miR-301-3p directly targets PTEN and TLR4 in the regulation of erectile function. However, there is a need to further investigate miR-301a-3p, PTEN, and TLR4 as possible therapeutic targets for treating ED. In this study, performing CIH-induced injury in cell experiments enabled the measurement of ICP/RT-AP and smooth muscle staining in SD rats. Due to a number of factors, like the function of PTEN-OE and TLR4-OE not being studied in SD rats, this study still needs further development.

## Conclusions

In summary, we found that miR-301a-3p might play an important role in the progression of post-RALP-related or CIH-mediated ED by targeting PTEN and TLR4 thereby affecting the expression levels of α-SMA, eNOS, cell autophagy, and apoptosis. Despite miR-301a-3p/PTEN and miR-301a-3p/TLR4 signaling pathway needing further investigation, our findings suggest that miR-301a-3p should be considered a new therapeutic target for ED treatment.

## Supplementary Information

**Additional file 1.**

## Data Availability

The data generated or analyzed during this study are included in this article, or if absent are available from the corresponding author upon reasonable request.

## Abbreviations

ED: Erectile dysfunction
OSA: Obstructive sleep apnea
ADSCs: Adipose-derived mesenchymal stem cells
miRNA: MicroRNA
CIH: Chronic intermittent hypoxia
CCSMCs: Corpus cavernous smooth muscle cells
PTEN: Phosphatase and tensin homolog
TLR4: Toll-like receptor 4
HIF-1α: Hypoxia-inducible factor-1 alpha
RALP: Robotic-assisted laparoscopic radical prostatectomy
CN: Cavernous nerve
nNOS: Neuronal nitric oxide synthase
eNOS: Endothelial nitric oxide synthase
PDE-5: Phosphodiesterase type 5
SD: Sprague Dawley
MSCs: Mesenchymal stem cells
OE: Overexpression
PBS: Phosphate-buffered saline
DMEM: Dulbecco’s modified Eagle’s medium
FBS: Fetal bovine serum
FCM: Flow cytometry
EDTA: Ethylenediaminetetraacetic acid
AP: Arterial pressure
ICP: Intracavernous pressure
MICP: Maximum ICP
RT-AP: Real-time carotid arterial pressure
NC RNAs: Non-coding RNAs
T2DMED: Type 2 diabetes mellitus-associated erectile dysfunction
NC: Negative control
miR-NC: miRNA mimic negative control
Exo: Exosomes from untreated ADSCs
Exo-301a: Exosomes from miR-301a-3p overexpressing ADSCs
DAPI: 4′,6-Diamidino-2-phenylindole
RT-qPCR: Quantitative reverse transcription polymerase chain reaction
TEM: Transmission electron microscope
α-SMA: Alpha smooth muscle actin
DNP: Dorsal nerve of the penis
iNOS: Inducible nitric oxide synthase
WT: Wild type
MUT: Mutant type
FITC: Fluorescein isothiocyanate
ns: Non-significant
NTA: Nanoparticle tracking analysis
PI: Propidium iodide
GFP: Green fluorescent protein
mRFP: Monomeric red fluorescent protein
