# Supplementary material for: Exosomes derived from miR-301a-3p-overexpressing adipose-derived mesenchymal stem cells reverse hypoxia-induced erectile dysfunction in rat models
Source: Stem Cell Res Ther. 2021 Jan 25;12:87. doi: 10.1186/s13287-021-02161-8 (PMC7836493; doi:10.1186/s13287-021-02161-8)
Supplement: Supplementary file 1 — Additional file 1. [file 13287_2021_2161_MOESM1_ESM.docx]

Exosomes derived from miR-301a-3p-modified adipose-derived mesenchymal stem cells reverse hypoxia-induced erectile dysfunction in rat models

Li Liang^b,1^, Dachao^a,1^ Zheng, Chao Lu^a^, Qinghong Xi^a^, Hua Bao^a^, Wengfeng Li^a^, Yufei Gu^a^, Yuanshen Mao^a^, Bin Xu^a^, Xin Gu^a^

*a* *Department of Urology, Shanghai Ninth People’s Hospital, Shanghai Jiao Tong University School of Medicine, 201999, China.*

*b Department of Respiratory Medicine, Shanghai Ninth People’s Hospital, Shanghai Jiao Tong University School of Medicine, Shanghai, 201999, China.*

* Corresponding authors

Xin Gu, PhD, Department of Urology, Shanghai Ninth People’s Hospital, Shanghai Jiao Tong University School of Medicine, 201999, China

E-mail addresses: guxin111@163.com

Bin Xu, PhD, Department of Urology, Shanghai Ninth People’s Hospital, Shanghai Jiao Tong University School of Medicine, 201999, China

E-mail addresses: chxb2004@126.com

^1^ These authors contributed equally to this work.

**Materials and methods**

Total RNA and miRNA isolation and quantitative reverse transcription-polymerase chain reaction (RT-qPCR)

RNA was isolated from rat tissues or CCSMCs using TRIzol reagent (Invitrogen, CA, USA). miRNA was isolated from human patients and rat’s serum using miRNeasy kits (Qiagen, Hilden, Germany) according to the protocol provided by the manufacturer. miScript II RT Kit was applied for reverse transcription and a miScript SYBR Green PCR Kit was applied for cDNA amplification. RT-qPCR was performed using a SYBR Premium Ex Taq II kit (TaKaRa, Dalian, China) on an ABI PRISM 7500 Sequence Detection System (Applied Biosystems). All reactions were performed in triplicate with the mean value being used to calculate expression levels after normalization against β-actin for mRNA and U6 for miRNA.

Protein extraction and Western blot analysis

Cytosolic and nuclear proteins in cells or tissues were isolated as described in NE-PER™ Nuclear and Cytoplasmic Extraction Reagents (Thermo Fisher, MA, USA) and the protein concentration determined using a BCA protein assay kit (Thermo Fisher). Approximately 30μg of protein from each sample was separated using 10% sodium dodecyl sulphate-polyacrylamide gel electrophoresis (SDS-PAGE) and transferred to a polyvinylidene fluoride (PVDF) membrane. Membranes were then blocked using 5% skim milk in TBST followed by incubation at 4°C overnight with one of the following primary antibodies: α-SMA (ab32575, 1:1,000), eNOS (ab76198, 1:1,000), nNOS (ab76067, 1:1,000) , iNOS (ab178945, 1:1,000), PTEN (ab32199, 1:1,000), HIF-1α (ab51608, 1:1,000), LC3B (ab48394, 1:1,000), p62 (ab109012, 1:1,000), TLR4 (ab22048, 1:1,000), p65 (ab16502, 1:1,000), LaminB (ab16048, 1:1,000), and β-actin (ab8226, 1:1,500) all from Abcam. Membranes were then incubated with the corresponding secondary antibody for 1 hour at room temperature and washed in TBST. Protein signals were detected using Super ECL Plus Detection Reagent (Merk).

Cell transfection

For miR-301a-3p overexpression, chemically synthesized miR-301a-3p mimic (Sense: 5’- CAGUGCAAUAGUAUUGUCAAAGC-3’, Anti-sense: 5’- GCUUUGACAAUACUAUUGCACUG-3’) and miRNA mimic negative control (miR-NC) (Sense:5’-UCACAACCUCCUAGAAAGAGUAGA-3’, Anti-sense: 5’-UCUACUCUUUCUAGGAGGUUGUGA-3’) was purchased from Hanbio Biotechnology Co., Ltd. ADSCs were transfected with either the miR-301a-3p mimic or mR-NC at a final concentration of 50 nmol/L using Lipofectamine 2000 (Invitrogen) following the protocol described by the manufacturer. After 48 hours of transfection, cells were used for the analysis of miR-301a-3p expression and other experiments.

For the overexpression of TLR4 and PTEN, the TLR4 and PTEN plasmid was constructed by subcloning the full-length complementary DNAs (cDNAs) of rat TLR4 and PTEN into the pcDNA3.1 vector. CCSMCs were transfected with TLR4 and PTEN vector at a final concentration of 50 nmol/L using Lipofectamine 2000 (Invitrogen) following the protocol described by the manufacturer. After 48 hours of transfection, cells were used for the analysis of miR-301a-3p expression and other experiments.

Nanoparticle tracking analysis (NTA)

The number and size of the exosomes were measured directly using a NanoSight LM10 nanoparticle tracking system (NanoSight Technology, Malvern, UK). Exosomes were resuspended in PBS at a concentration of 5 μg/mL and further diluted 100- to 500-fold to achieve 20–100 objects per frame. Samples were injected manually into the sample chamber at ambient temperature. Each sample was configured with a 488-nm laser and a high-sensitivity camera, and monitored in triplicate at a camera setting of 13 with an acquisition time of 30 s and a detection threshold setting of 7. At least 200 completed tracks were analyzed per video. The data were finally analyzed using NTA analytical software (version 2.1).

Luciferase reporter assay

To construct luciferase reporter vectors, the 3ʹ-UTR of TLR4 and PTEN cDNA fragments containing the predicted potential miR-301a-3p binding sites were amplified by PCR and sub-cloned downstream of the luciferase gene in the pmirGlo Dual-Luciferase vector (Promega, Fitchburg, WI, USA). CCSMCs cells were cultured in 24-well plates and co-transfected with 50 ng of the corresponding vectors containing firefly luciferase together with 25 ng of miR-301a-3p or the control. Transfection was performed using Lipofectamine 2000 reagent (Invitrogen). At 48 h post-transfection, relative luciferase activity was calculated by normalizing the Firefly luminescence to the Renilla luminescence using a Dual-Luciferase Reporter Assay (Promega, Fitchburg, WI, USA) according to the manufacturer’s instructions.

Flow Cytometry Assay

CCSMCs cells were seeded into culture flasks. The cells were dual stained with Annexin V-FITC and propidium iodide (PI) for 30 min at room temperature. The stained cells were immediately analyzed by flow cytometry (Becton Dickinson, Franklin Lakes, NJ, USA). Apoptotic cells are defined by Annexin V-FITC positive and PI negative staining.

Autophagic flux analysis

CCSMCs were transfected with mRFP-GFP-LC3 adenoviral vectors (HanBio, Shanghai, China) for 24 hours. After transfection, mRFP-GFP-LC3-CCSMCs cells were fixed with 4% paraformaldehyde and stained using 10 μmol/L Hoechst 33 342. An Operetta High Content Imaging System (Perkin-Elmer) was used to obtain the images of the cells which were analyzed using Harmony analysis software (Perkin-Elmer). Green fluorescent protein (GFP) or monomeric red fluorescent protein (mRFP) were used for cell detection. In merged images, puncta in autophagosome and autolysosomes stained yellow and red respectively. Autophagic flux was determined by the increased percentage of red puncta in merged images.

Transmission electron microscopy (TEM)

Fixation of CCSMCs was done using 2.5% glutaraldehyde in PBS followed by storing at 4°C. Post-fixation of the cells was done using 1% osmium tetroxide followed by an increasing gradient dehydration step using ethanol and acetone. Cells were then embedded in Araldite followed by sectioning where ultrathin sections were obtained 50‐60 nm. Obtained sections were placed on uncoated copper grids and stained using 3% lead citrate-uranyl acetate. Images of the sections were captured using a CM-120 electron microscope (Philips).
